# Challenges and Limitations of Biological Network Analysis

**DOI:** 10.3390/biotech11030024

**Published:** 2022-07-07

**Authors:** Marianna Milano, Giuseppe Agapito, Mario Cannataro

**Affiliations:** 1 Department of Medical and Clinical Surgery, University Magna Græcia, 88100 Catanzaro, Italy; m.milano@unicz.it (M.M.); cannataro@unicz.it (M.C.); 2 Data Analytics Research Center, University Magna Græcia, 88100 Catanzaro, Italy; 3 Department of Law, Economics and Social Sciences, University Magna Græcia, 88100 Catanzaro, Italy

**Keywords:** biological pathways, protein–protein interaction, networks alignment, local and global alignment

## Abstract

High-Throughput technologies are producing an increasing volume of data that needs large amounts of data storage, effective data models and efficient, possibly parallel analysis algorithms. Pathway and interactomics data are represented as graphs and add a new dimension of analysis, allowing, among other features, graph-based comparison of organisms’ properties. For instance, in biological pathway representation, the nodes can represent proteins, RNA and fat molecules, while the edges represent the interaction between molecules. Otherwise, biological networks such as Protein–Protein Interaction (PPI) Networks, represent the biochemical interactions among proteins by using nodes that model the proteins from a given organism, and edges that model the protein–protein interactions, whereas pathway networks enable the representation of biochemical-reaction cascades that happen within the cells or tissues. In this paper, we discuss the main models for standard representation of pathways and PPI networks, the data models for the representation and exchange of pathway and protein interaction data, the main databases in which they are stored and the alignment algorithms for the comparison of pathways and PPI networks of different organisms. Finally, we discuss the challenges and the limitations of pathways and PPI network representation and analysis. We have identified that network alignment presents a lot of open problems worthy of further investigation, especially concerning pathway alignment.

## 1. Introduction

High-Throughput Omics (HTO) experimental platforms that include protein, Single-Nucleotide polymorphisms (SNPs) and gene expression microarrays, Genome-Wide Association Studies (GWAS) and Next-Generation Sequencing (NGS) can simultaneously investigate thousands of genes for a single experiment. In addition, experimental techniques such as yeast two-hybrid (Y2H) and mass spectrometry (MS), are commonly used to detect interacting proteins. HTO platforms promoted the holistic theory that both complex and common diseases are due to the interactions of several muted genes or proteins, contrasting with the old theory that a disease is due to the mutation of a single gene [1]. In this landscape, the main problem was to define a model that can effectively represent interactions among genes and proteins. In general, different biological systems are modeled according to graph-theory formalism, which enables us to represent the entities of a system as nodes and their relations as edges. For instance, Protein–Protein Interaction (PPI) Networks model biochemical interactions among proteins [2]. Nodes represent the proteins from a given organism, and the edges represent the interactions among proteins. The representation as a graph is convenient for a variety of reasons. Networks provide a simple and intuitive representation of heterogeneous and complex biological processes. Additionally, the graph formalism enables us to conduct network-based analysis. A common analysis regarding the comparison of graphs is based either on comparing their global properties, such as their clustering coefficient or node degree distribution, or the analysis of their internal structure, formally known as network alignment (NA). For instance, the comparison of PPI networks, based on NA, has evidenced the conservation of patterns of interactions among the evolution [3]. Instead, in biological pathways, the nodes represent proteins, RNA and fat molecules, for example, while the edges represent the interaction between molecules. It is notable that, since the pathways are classified in metabolic, signaling and regulatory ones, this definition does not hold for metabolic pathways. Biological pathways consist of proteins, RNAs and other molecules connected by interactions such as activation or catalysis. For example, the trigger of interactions among biological entities in cells produces new molecules (e.g., fat or protein). This is more straightforward if expressed in a visual format. Biological pathways simplify the description, representation and modeling of several biological events such as reactions, phosphorylation, catalysis, inhibition, deactivation, etc., that govern the biochemical machinery normal or abnormal cell cycle. Using a graphical or textual model to represent biochemical events makes it easier to share information, study and understand these complex events [4].

Using graphs to model pathways and PPI networks has made it possible to organize, store and exchange pathway information, promoting the development of pathway databases containing the relevant experimental and electronically inferred evidence information. On the other hand, problems related to the integration, visualization and representation of such massive networks arise, spurring computer scientists to develop new efficient algorithms to deal with these vast networks [5].

The remaining part of the manuscript is arranged as follows. Section 2 presents the main data models used to represent pathways and PPIs. Section 3 introduces pathways and PPI formats for integration, exchange, visualization and analysis. Section 4 and Section 5 provide a review of some well-known pathway and PPI databases. Section 6 provides a review of the NA algorithm available for PPI and pathway, highlighting their strengths and weaknesses. Section 7 discusses the differences between PPI and pathway NA algorithms. Finally, Section 8 concludes the manuscript and delineates some possible solutions to the NA algorithm open problems.

## 2. Background

In this section, we present the available data models to represent pathways and PPI.

### 2.1. Pathway Data Models

Pathways can be classified in three main classes: *Metabolic*, *Gene Regulation* and *Signal Transduction*.

- *Metabolic Pathways* represent chemical reactions carried out by a cell to transform food into energy, for example.
- *Gene Regulation Pathways* are responsible for the activation (or inhibition) of genes (e.g., on, off).
- *Signal Transduction Pathways* govern the transmission of signals from a source to a destination.

This classification can be helpful in the process of discovering new pathways or functions. Indeed, identifying the constituents involved in a pathway provides clues about the pathway working principles and, thus, the pathway type. Thus, representing pathways as a network enables the investigation of global structures instead of the behavior of individual molecules, making it possible to highlight and develop a remarkable understanding of how these complex networks work.

To represent a pathway as a network, it is possible to use graph theory. A graph is a pair G=(V,E) consisting of a set of vertices *V* and a set of edges *E*, where an edge connects two vertices. An edge e∈E connecting the vertices u,v∈V is denoted by {u,v}∈E, and *u* and *v* are called adjacent (or neighbors) and they are incident with *e*. This definition describes undirected graphs; that is, graphs where connections between vertices are without a direction. Instead, in directed graphs, the two edges are different $e_{1}=\left\{u,v\right\}\neq e_{2}=\left\{v,u\right\}$; thus, the connections between vertices have a direction. Often, this needs to extend graph models by adding attribute to the vertices and edges, such as text, numerical values, types, colors and coordinates. Typical examples include the stoichiometry of reactions in metabolic pathways represented as numerical values on the edges. To assign attribute to edges or nodes, it is possible to use a mapping function Π. The mapping function Π maps an attribute type to the vertices or edges. Thus, a stoichiometry value can be represented as a numeric value and mapped to an edge, e.g., a real value connecting two vertices. The Π function can be formally defined as: Π:ℜ→E, that assigns at each edge *e* a weight Π(e).

Due to the different type of biological pathways with different properties, the graph models have to be tailored to the specific network under consideration. Thus, graphs can be classified as *directed*, *undirected*, *mixed*. Undirected graphs represent PPI networks where interactions (edges) are without a direction. In an undirected graph, an edge between the vertices *u* and *v* is an unordered vertex pair {u,v}∈E. Conversely, directed graphs are suitable to model metabolic, signaling and gene-regulation pathways. In a directed graph, an edge between the vertices *u* and *v* is an ordered vertex pair {u,v}∈E≠{v,u}∈E. In a mixed graph, both undirected and directed edges are present. Mixed graphs are also relevant in pathways representation, where some interactions are undirected and others are directed, such as, for instance, activation and phosphorylation.

*Multigraphs* are graphs holding multiple edges; that is, two or more edges between the same two vertices, and in the case of directed graphs, they could have different directions. In a multigraph, such edges are also called multiedges. Multiedges are, useful for the modeling of metabolic pathways, where the same compounds can be transformed by different reactions.

*Hypergraphs* are an extension of graphs and multigraphs. *Hypergraphs* enable the representation of metabolic reactions in pathway networks, where several compounds react together to catalyze new products. For instance, in signaling pathways, several molecules interact among them to control one or more small molecules to transport the signal from inside to the external of nucleus. Meanwhile, in regulatory pathway, hypergraphs can effectively show the interactions of multiple biological entities necessary to activate or deactivate the gene’s activity. A hypergraph H=(V,E) consists of a set of vertices *V* and a set of hyperedges *E*, where each hyperedge is a non-empty subset of *V*. Hypergraphs can be directed or undirected. Hypergraphs are not commonly used in graph theory, but such graphs are used to model biological networks, especially pathways.

### 2.2. Protein–Protein Interaction Data Models

Protein–protein Interaction (PPI) Networks represent the interactions among proteins [6]. PPI networks are fundamental for cellular functions; for example, the assembly of cell structural components or transcription, such as processes translation and active transport. PPI networks are modeled by using graphs. In general, PPIs are represented as directed or undirected graphs, where the nodes represent the proteins and the edges correspond to the interactions among connected proteins. Clearly, this simple representation does not capture the following aspects of interaction: the kind of interaction itself, e.g., phosphorylation or complexation or colocalization, and some other particular aspects strictly tied to the kind of reaction, such as the direction or the kinetics. If one wants to provide a distinction between reagent and product, or one wants to represent biochemical reactions, a bit of complexity is necessary. In this case, a directed graph will represent the distinction between reagents and products. Finally, a label on the edges can specify the kind of interaction, i.e., phosphorylation, alkylation, ubiquitination. The model based on directed graphs can be similarly used to model metabolic reactions. In this case, nodes can be proteins, nucleic acids, compounds or metabolites, and edges represent all kind of interactions. The determination of a correct model for PPI networks could be important for effective experimental planning, helping to determine possible interactions. Currently, there are three common models used for PPI networks: Random Graph, Scale Free and Geometric Random Graph [7]. Starting from the highest abstract level, the components of interactions (e.g., proteins and enzymes) can be modeled as a set of nodes connected by edges representing the interactions. This informal model is easily traduced in a graph by using a formal mathematical language.

The Erdos–Renyi model [8] is an abstract representation of a random network in which a specified probability describes the existence of an edge between each couple of nodes.

Formally, a *random graph*, G(n,p) is a graph with *n* nodes, where each possible edge has probability *p* of existing. Consequently, the number of edges in such a graph is a random variable. G(n,p) can be seen as a set of graphs with *n* nodes, in which each graph is denoted by its probability related to its number of edges. For a random graph, the average degree *z* of a vertex is equal to

(1) $$z=\frac{n(n-1)}{n}\approx np$$

for large number of *n*. So, once one knows *n*, any property can be expressed both in terms of *p* or *z*. Consequently, this model presents the advantage of summarizing the topological properties in two parameters, *n* and *p*. Briefly, it is possible to recall that for large values of *n* (or alternatively, when z=1), random graphs exhibit a transition phase causing the formation of a so-called giant component. A component is a subset of nodes, which are all reachable from other nodes. A giant component, consequently, is the largest component. The formation of a giant component is a characteristic of many real networks, both biological and social. Despite this, random graphs do not capture the high clustering coefficient property of real networks. This drawback also appears in metabolic networks, as reported in [9]. In that work, authors analyze a metabolic network of *E. coli* by building a graph of interactions in which vertices represent substrates and products and edges represent interactions. The clustering coefficient of the network is 0.59 while a random graph with the same number of node presents a value of 0.09.

The main characteristic of scale-free networks [10] is the power-law degree distribution; that is, the probability that a generic node has exactly *k* edges, that is expressed by $P\left(k\right)=k^{\gamma }$, where γ is the degree exponent. A property of these networks is the presence of a small number of highly connected nodes (called *hubs*) which determine other properties. Generally, for these networks, the clustering coefficient is independent of the number of nodes *n* and the diameter is very small, following the d≃loglog(n) formula.

*A geometric graph* [11] G(V,r) is a graph whose nodes are points in a metric space which are connected by an edge if their distance is below a threshold value *r*, called radius. Formally, let u,v∈V; the edge set is E={{u,v}|(u,v∈V)∧(0<∥u−v∥<r)}, where ∥.∥ is a defined distance norm. Generally, a two dimensional space is considered, and norms are the well known Manhattan or Euclidean distance and the radius takes values in (0,1).

Thus, a *random geometric graph* G(n,r) is a generalization of a geometric graph in which nodes correspond to *n* points in a metric space. Clearly, these points are distributed uniformly and independently. The properties of these graphs have been studied when n→∞ [11]. Surprisingly, certain properties of these graphs appear only when a specific number of nodes is reached.

## 3. Standards for Pathway and PPI Networks Encoding

Biologists need to use information from many sources to support their research, and this led to an increase in the number of pathway databases. On the other hand, each database adopted different representation data formats, making data integration and exchange between multiple databases a challenge. Thus, the need to establish an unique pathway representation format that can integrate data from various databases arises. Pathway data are encoded using several formats such as: *BIOPAX-LEVEL 1,2,3*-, *CellML*, *HUPO*, *PSI-MI* and *SBML*, as described below.

### 3.1. BioPAX

BioPAX (Biological Pathway Exchange) [12] aims to simplify the integration and exchange of data stored in biological pathway databases through a unique way to represent data. BioPAX defines pathway data through the *OWL DL* meta-language and represented in the *RDF/XML* format. Traditionally, the integration and comparison of different data formats has always been a challenge in bioinformatics. Thus, BioPAX purses the goal of simplifying data integration, comparison and exchange by using a unique common format. BioPAX Level 3 is the recommended version, since it supports the representation of metabolic pathways, signaling pathways (including states of molecules and generic molecules), gene regulatory networks, molecular interactions and genetic interactions. BioPAX Level 3 is not fully backward compatible with Levels 1 and 2.

### 3.2. CellML

CellML [13] is an XML-based markup language for describing, storing and exchanging computer-based mathematical models. CellML allows scientists to share models even if they are using different model-building software, to reuse components from one model into another one, accelerating model building. CellML allows the description of biological models, including information about the model structure, equations describing the underlying processes and additional information, simplifying the search of specific models or model components in a database. The CellML provides a comprehensive word-list describing biological information with different resolutions, starting from the sub-cellular to the organism level.

### 3.3. HUPO

HUPO PSI Molecular Interactions Tab-Delimited format (PSI-MITAB) The authors of [14] introduce a tabular data exchange format suitable for describing interactions between biological entities. PSI-MITAB improves the annotations and representations of molecular interaction data and the exchange of molecular interaction data to the user community. Thus, data stored in multiple databases can be downloaded and easily integrated.

### 3.4. PSI-MI

PSI-MI (Proteomics Standards Initiative—Molecular Interaction) [15] format provides an XML standard for molecular interactions and is supported by many molecular interaction databases and tools. The PSI-MI format defines a standard for data representation in proteomics to facilitate data comparison, exchange and verification, and it is not a proposed database structure.

### 3.5. SBML

SBML (Systems Biology Markup Language) [16] is an XML-based language used to represent, store and exchange models of biological systems of varying complexity. SBML offers a collection of components and features fundamental for describing a wide range of dynamical models, simulated by using ordinary differential equations (ODEs) and stochastic formalism. The ongoing development of SBML contributed to enhancing its extensibility, portability and re-usability, for describing whole-cell models. SBML levels are intended to coexist. Thus, the definition of a new level does not make the previous one obsolete, since it introduces new functionalities, constructs and features, enhancing analyses and simulations performance.

### 3.6. PPI Networks

In proteomics, the description of the set of protein–protein interactions remains one of the main objectives. In fact, several experiments both on a small and large scale have made it possible to shed light on the different nature of interaction networks. In this context, the necessary data integration between experiments is currently hampered by the fragmentation of public availability protein interaction data, which exist in different formats in databases, on corporate and academic websites or sometimes just in scientific publications. In general, there are several consolidated databases for protein interactions (which will be covered in a dedicated section), which mainly use two standard data models for the representation and exchange of protein interaction data. These standards are PSI-MI [15] and HUPO [14]. For a complete description, see above.

## 4. Pathway Databases

Each database organizes its pathways data in networks to provide insight into the affected biological functions underlying the cellular mechanisms. Although this division into pathways is not arbitrary and is based on physical criteria in each database, there is no generally accepted pathway definition. The description of the main characteristics of some well-known pathway databases can be seen below.

*Biocarta* (https://www.hsls.pitt.edu/obrc/index.php?page=URL1151008585) [17] is a database of maps representing metabolic pathways, signal transduction pathway, and other biochemical pathways. Pathway data can be downloaded by using the provided Rest application programming interface (API) or through the Harmonizome web-portal. A REST API, also known as a RESTful API, is an application programming interface that conforms to the constraints of the REST architectural style, enabling interaction with RESTful web services. The term REST, coined by computer scientist Roy Fielding, stands for REpresentational State Transfer. Biocarta provides information on 254 human and mouse pathways collected from 66 online sources.

*BioCyc* (https://biocyc.org) [18] is a collection of 20,005 Pathways and Genome Databases (PGDBs) for model eukaryotes and for thousands of microbes. BioCyc is an encyclopedic reference that contains curated data from about 130,000 publications. BioCyc integrates genome data with additional data including metabolic reconstructions, regulatory networks, protein features, orthologs, gene essentiality and atom mappings. In additon, BioCyc provides several software tools for data analysis. BioCys requires a paid subscription to access data, whereas the EcoCyc and MetaCyc databases are freely available.

*INOH* (Integrating Network Objects with Hierarchies) (https://dbarchive.biosciencedbc.jp/en/inoh/desc.html) [19] provides information on the molecular pathways in humans, mice, rats and other organisms. INOH collects pathway data from the literature. INOH pathway’s terms are enriched with information from at least two additional databases, SWISS-PROT [20] and Gene Ontology (GO) [21]. Pathway data are freely available to download for later use in BioPAX, OBO and INOH formats.

*KEGG* (Kyoto Encyclopedia of Genes and Genomes) (https://www.kegg.jp/kegg/) [22] is an integrated database encompassing 16 databases, providing systems information, genomics information, chemical information and health information. Pathways in KEGG are manually drawn as maps representing knowledge about metabolism, cellular process, human diseases, drug development, genetic information processing and environmental information processing. KEGG pathway data can be visualized through a web browser and accessed by using KEEG API available only for academic use; for other purposes, the download requires a licence.

*NetPath* (http://www.netpath.org) [23] is a manually curated resource of signal pathways. In the current version, NetPath contains 36 curated human pathways. NetPath pathways are freely available under an adaptive Creative Commons License for download as BioPAX level 3.0, PSI-MI version 2.5 and SBML version 2.1 formats.

*PathwayCommons* (https://www.pathwaycommons.org) [24] is a collection of public pathway databases, such as Reactome, PID and Cancer Cell Map, as well as protein–protein interaction databases, such as HPRD, HumanCyc, IntAct and MINT. The main goal of PathwayCommons is to provide an access point for a collection of public databases, and it includes technology for integrating pathway information. Pathway creation, extension, and curation remain the duty of the source pathway databases. PathwayCommons provides a web interface to browse pathways, as well as a web service API for automatic access to the data. Additionally, PSI-MI and BioPAX formats are supported for the data download. Furthermore, the complete PathwayCommons database can be automatically accessed using the PathwayCommons plugin.

*Reactome* (https://reactome.org/) [25] is a manually curated and peer reviewed repository of signaling and metabolic pathways and processes. In the current version, Reactome contains pathways for 15 different organisms, including the *Homo sapiens*. Reactome includes 2553 pathways and 11,071 annotated proteins for the *Homo sapiens*. All pathway data are freely available for download in the following formats: Neo4j GraphDB, MySQL, BioPAX, SBML and PSI-MITAB files. They are also accessible through the Reactome Web Services APIs.

*SMPDB* (Small Molecule Pathway Database) (https://www.smpdb.ca) [26] is an interactive visual database containing about 30,000 small molecule pathways discovered in humans. SMPDB is explicitly designed to support pathway elucidation and discovery in metabolomics, transcriptomics, proteomics and systems biology. SMPDB pathways include information on the relevant organs, subcellular compartments, protein complex cofactors, protein complex locations, metabolite locations, chemical structures and protein complex quaternary structures. SMPDB supports full searching through lists of metabolite names, drug names, gene names, protein complex names, SwissProt IDs and GenBank IDs. Result search produces lists of matching pathways and highlights the matching molecules on each pathway diagram. SMPDB’s images, maps, descriptions, and tables are freely available for download.

*WikiPathways* (https://www.wikipathways.org/) [27] is an open, collaborative platform for the curation of biological pathways. WikiPathways is a new model of pathway databases that improves and complements ongoing efforts, such as KEGG, Reactome and PathwayCommons. WikiPathways has a dedicated web page, displaying diagrams, description, references, download options, version history and component gene and protein lists. WikiPathways is freely available for download in the form of images, and in GPML, or in a custom XML format. In addition, data can be accessed programmatically by using the available Webservice/API.

The main features of the listed pathway databases are summarized in Table 1.

## 5. Protein–Protein Interaction Databases

The increase in high-throughput experiments and the development of computational prediction methods to discover protein–protein interactions have led to the introduction of several publicly available PPI databases. In general, the PPI databases can be distinguished in two kind of classes: databases of experimentally determined interactions, such as DIP [28], BioGRID [29], MINT, [30], Intact [31], that store interactions extracted from both literature and high-throughput experiments, and databases that store predicted interactions obtained using in silico methods, such as I2D [32], STRING [33].

The first class of databases contain the data of experimentally determined interactions. These databases go beyond the storing of the interactions and integrate them with functional annotations, sequence information and references to corresponding genes. They also enable some visualization that presents a subset of interactions in a comprehensive graph.

*DIP* (Database of Interacting Proteins) (https://dip.doe-mbi.ucla.edu/dip/Main.cgi) contains interactions experimentally determined in different organisms, such as Saccharomyces cerevisiae, Drosophila melanogaster, Escherichia coli, Caenorhabditis elegans, Homo sapiens, Helicobacter pylori, Mus musculus, Rattus norvegicus, Bos taurus and Arabidopsis thaliana. Currently, the database contains 81,923 interactions of 28,850 different proteins obtained from 82,143 distinct experiments. The interaction data in DIP have been revised both by expert curators and by computational approaches that apply prior knowledge about the protein–protein interaction networks.

DIP can be searched through its web-based interface, which allows users to query for a protein. Various subsets of the DIP interaction data are available in a variety of formats such as PSI-MI, MITAB2.5 (tab-delimited format) and the legacy XIN format (a legacy XML format that was used before introduction of PSI-MI (MIF)). The sequences of the proteins participating in DIP interactions are provided in FASTA format.

*BioGRID* (Biological General Repository for Interaction Datasets) (https://thebiogrid.org/) is a biomedical interaction repository. The current version contains 2,467,140 proteins and 1,740,000 interactions curated from both high-throughput datasets and individual focused studies, as derived from over 70,000 publications in the primary literature, from major different model organism species (Arabidopsis thaliana, Bos taurus, Caenorhabditis elegans, Chlorocebus sabaeus, Cricetulus griseus, Danio rerio, Drosophila melanogaster, Gallus gallus, Homo sapiens, Macaca mulatta, Mus musculus, Rattus norvegicus, Saccharomyces cerevisiae, Severe acute respiratory syndrome coronavirus 2 and Xenopus laevis). The query interface of BioGRID is based on a web interface that enables searches by inserting protein or gene identifiers as well as article identifiers or publication keywords. The whole set of BioGRID data may be downloaded in multiple file formats, including PSI-MI XML.

*MINT* (Molecular Interaction database) (https://mint.bio.uniroma2.it/) is designed to store data on functional interactions between proteins. The current version of the database contains 132,249 interactions and 27,069 proteins from Homo sapiens, Saccharomyces cerevisiae, Mus musculus, Rattus norvegicus, Helicobacter pylori, Drosophila melanogaster and Caenorhabditis elegan organisms. This database does not contain only physical interactions between proteins, but also, it is conceived to store other kinds of molecules (e.g., enzymes or nucleic acids). The database can be accessed through a web interface by inserting the protein name, the accession number or other identifying keywords. Results are presented in in HUPO PSI-MI XML and PSI-MI TAB format.

*IntAct* (https://www.ebi.ac.uk/intact/home) database is a repository of interactions and is completely based on open-source software. It contain 118,759 proteins and 1,184,144 interactions from Homo sapiens, Saccharomyces cerevisiae, Mus musculus, Rattus norvegicus, Escherichia coli, Drosophila melanogaster, Caenorhabditis elegan, Helicobacter pylori, Schizosaccharomyces pombe, Bacillus subtilis and SARS-CoV-2 model species organisms. The query interface of BioGRID is based on a web interface that enables searches by inserting proteins or gene identifiers in the UniProt ACs, Taxon IDs, Publication IDs, Complex ACs, GO terms formats. The BioGRID data may be downloaded in multiple file formats, including PSI MI XML.

The second class of databases contain the predicted interactions starting from existing databases of verified interactions. Different algorithms have been developed to predict putative interactions. These algorithms work on verified interactions datasets by storing biological information in order to produce a set of putative interactions. For example, the algorithms use orthologs information, i.e., the information that the interaction mechanisms are conserved through the evolution, and starting from two interacting proteins in a organism, e.g., A and B, they find the orthologs of A and B in other organisms.

For example, *I2D* (Interologous Interaction Database) (http://ophid.utoronto.ca/ophidv2.204/) is an online database of known and predicted mammalian and eukaryotic protein–protein interactions. It has been built by mapping high-throughput data between species. Thus, until experimentally verified, these interactions should be considered predictions. It remains one of the most comprehensive sources of known and predicted eukaryotic PPIs. The prediction algorithm is based on the hypothesis of conservation of patterns of molecular interaction through the evolution. On the basis of such consideration, a model for mapping interactions of model organisms into humans has been developed. I2D is searchable via a web interface. The latest release of I2D contains 687,072 proteins and 1,279,157 (predicted and source) interactions, and it includes data for *S. cerevisiae*, *C. elegans*, *D. melonogaster*, *R. norvegicus*, *M. musculus* and *H. sapiens*. I2D can be queried by using single or multiple protein IDs. Results can also be visualized using its graph visualization program. A freely downloadable software tool called NAViGaTOR [34,35] (Network Analysis, Visualization, Graphing TORonto) queries the I2D database and visualizes networks. Data can be exported both in tab-delimited and PSI-MI formats.

*STRING* (Search Tool for the Retrieval of Interacting Genes/Proteins database) (https://string-db.org/) is a database of predicted interactions for different organisms such as Homo sapiens, Mus musculus, Arabidopsis thaliana, Saccharomyces cerevisiae, Escherichia coli, Caenorhabditis elegans, Rattus norvegicus, Drosophila melanogaster, Bacillus subtilis and Pseudomonas aeruginosa PAO1. The database can be accessed on the website by specifying a protein identifier, or alternatively, by inserting the protein primary sequence. The current version contains 67,592,464 proteins and 20,052,394,041 total interactions. The prediction algorithm is based on the concept of functional association. It considers conserved genomic neighbourhood, gene fusion events and co-occurrence of genes across genomes, as well as information about orthologs. The user can also download the primary data and the predictions as flat files or PSI- MI files, which cover selected views or the whole database. Table 2 summarizes the main characteristics of the listed databases.

Finally, the high level of heterogeneity among available databases contributes to limit the effectiveness of automatic data analysis.

## 6. Methods for the Analysis of Pathways and PPIs

### 6.1. Alignment of Biological Pathways

Similar to the alignment of sequence and structure, the alignment of pathways is obtained through a general optimization strategy that minimize differences among paired entities. Differences are defined in terms of both nodes and edges. Consequently, at the end of the alignment process, the closeness between two pathways is reflected by a score computed through a similarity function relying on a scoring model.

Alignment can be classified in two main groups: local alignments that maximize the similarity among local structures, and global alignment that maximizes the total number of corresponding nodes. More recent comparative research focuses on alignment techniques that can identify similar parts between pathways.

More formally, given two pathways $P_{1}$, $P_{2}$, the pairwise comparison and alignment of pathways is obtained by representing pathways as two directed hyper-graphs $DHG_{1}=\left\{V_{1},E_{1}\right\}$, $DHG_{2}=\left\{V_{2},E_{2}\right\}$, where *V* is a finite set of nodes and *E* is a set of directed hyper-edges. A directed hyper-edge is an ordered pair of subsets of nodes E=(X,Y), where *X* is the set of input nodes of *E*, while *Y* is its set of output nodes. The local alignment can be defined as a matching function $\pi :\left\{v\in V_{1}\right\}\to \left\{w\in V_{2}\right\}$, where π reflects the alignment of of a sub-set of pathway compounds (nodes in $V_{1}$ and $V_{2}$). Conversely a global alignment is a complete matching among all the nodes of two hyper-graphs, i.e., $\Pi :V_{1}\to V_{2}$. Recently, the traditional one-to-one matching was extended to one-to-many matching.

Here, we recall some well-known approaches for pathway alignments.

*MP-Align* [36] provides a methodology for the pairwise comparison and alignment of metabolic pathways, aiming to provide the largest conserved substructure of the pathways under consideration. MP-Align computes the alignment by means of the maximum weighted bipartite matching algorithm and the largest conserved sub-hypergraph methods. The method relies on five main phases. The first phase regards the reaction paths computation that is a reaction’s sequence, e.g., $P_{1}$ and $P_{2}$. The second phase establishes the first correspondence between $P_{1}$ and $P_{2}$. The third phase refines the correspondence between $P_{1}$ and $P_{2}$, defining a match called σ. The fourth phase translates the reaction path matching σ into a well-defined match between reactions in $P_{1}$ and $P_{2}$. The fifth phase determines the similarity score and the alignment of the two given pathways. MP-Align is implemented in Python, and it is freely available to download.

*PathAligner* [37] performs alignment by mapping the coordinates of one pathway onto the coordinates of one or more other pathways. PathAligner uses metabolite matching and EC number alignment to analyze the similarities between metabolic pathways. PathAligner assesses the alignment through a hierarchical alignment method.

*SubMAP* (Subnetwork Mappings in Alignment of Pathways) [38] extends the classical one-to-one mapping to the one-to-many mapping among the molecules (nodes) of the input graphs. SubMAP evaluates the alignment through the maximum weighted independent set and the eigenvalue problem methodologies. The method is based on a two-step strategy. SubMAP initially measures the similarities among nodes using a combination of genetic and topological information. Then it merges the two metric using a single scoring function. Finally the alignment is evaluated between two input pathways to maximize a similarity score, evaluated as the sum of the similarities of the mapped sub-networks of size *k*, excluding conflicting mappings. SubMAP is based on the maximum weight independent set (MWIS) problem.

*MPH* (MetaPathwayHunter) [39] is a pathway alignment tool that, given a query pathway and a pathway database, finds and reports all approximate occurrences of the query in the collection, ranked by similarity and statistical significance. MetaPathwayHunter figures out the alignment by means of the weighted assignments in bipartite graphs and the subtree homeomorphism methods.MPH relies on graph matching to resolve the labeled graph isomorphism problem. MPH is implemented by using a combination of C++ and Java code.

*MetaPAT* (Metabolic pathway alignment) [40] is able to perform the alignment of metabolic pathways and it does not restrict the topology of the network in any way. Instead, it exploits a property of metabolic networks known as ’local diversity property’. MetaPAT calculates the alignment by using the subgraph isomorphism method. MetaPATH has been implemented in C++.

*MetNetALigner* [41] aligns metabolic networks, taking in account the similarity of network topology and the enzymes’ functions. MetNetAligner allows users to predict unknown pathways, compare and find conserved patterns and resolving ambiguous identification of enzymes. The tool supports several alignment options such as allowing or forbidding enzyme, deletion and insertion. MetNetAligner models alignment as a dynamic programming whose solution is the alignment.

*CAMPways* (Constrained Alignment of Metabolic Pathways) [42] aligns pair of matabolic pathways. As a result, it provides one-to-many alignments of reactions in a pair of metabolic pathways, corresponding to a mapping between similar sub-structures of the pair. CAMPways implements an improved and effective version of the constraint alignment. CAMPways computes alignment solving the constraint alignment problem. Constraint alignment, even in a very primitive setting, is computationally intractable. CAMPWays provides better alignment results on metabolic networks compared to the state-of-the-art alternatives. CAMPWays is implemented by using C++ LEDA.

Table 3 summarizes the available types of pathway alignment algorithms.

### 6.2. Alignment of PPI Networks

A most promising method for the analysis of PPI networks to infer knowledge is Network Alignment (NA). Network alignment (NA) is a computational technique widely used for comparative analysis of PPI networks to discover evolutionarily conserved substructures among different species. Network alignment relates to the collection of methods that aim to find similarities among networks. The problem of graph alignment consists of the mapping between two or more networks to maximize cost function that represents the similarity among nodes or edges. Conventionally, given two graphs, $G_{1}=\left\{V_{1},E_{1}\right\}$ and $G_{2}=\left\{V_{2},E_{2}\right\}$, the graph alignment searches an alignment function $f:V_{1}\to V_{2}$, where $V_{1,2}$ are set of nodes, that maximizes the similarity between mapped nodes. Therefore, the graph alignment problem is based on the subgraph isomorphism issue, and for this, it results computationally NP-hard [43], and it should be resolved by heuristic methods. The alignment quality is a function of cost that measures the level of similarity of the analyzed network, and it is defined as follows: $Q(G_{1},G_{2},f)$. Q conveys the correspondence among the input networks on a precise alignment *f*. So, the Q definition highly affects the mapping approach [44].

In general, network alignment can be classified as Local Network Alignment (LNA) and Global Network Alignment (GNA). LNA algorithms usually aim to find regions of similarity between two or more networks by producing a many-to-many node mapping, and they are applied for the comparison of small regions extracted from two or more input networks.

These subnetworks are conserved patterns of interaction that can correspond to preserved or activity patterns. LNAs employ a two-step schema to build the alignment. At first, they take as input a set of seed nodes chosen by biological information; then, the algorithms merges the inputs in a complementary structure, also called an alignment graph. Finally, LNAs mine the alignment graph to extract interesting modules. Over the years, different LNAs algorithms have been implemented.

An example of the most recently developed algorithm is GLAlign (Global Local Aligner).

*GLAlign* (Global Local Aligner) is a novel LNA methodology [45] that first applies a GNA to generate a list of seed nodes on the basis of topological information. GLAlign computes the alignment through Seed nodes and Alignment Graph methods. Then, GLAlign integrates this topology information with biological information (i.e., homology relationships) by using a linear combination schema. At the end, GLAlign takes as input the generated global to compute a LNA.

*AlignNemo* [46] ensures the detection of sub-graphs of proteins with similar biological function according to the topology. AlignNemo assesses the alignment through a suitable Score Function. AlignNemo can build the alignment of sparse PPI networks because it analyzes the topology of adjacent nodes of interacting proteins.

*AlignMCL* [47] is the evolution of the previous AlignNemo. AlignMCL constructs the local alignment by integrating all the input data in the alignment graph, which is subsequently clustered by using MCL algorithm [48] to mine the conserved subnetworks. AlignMCL computes the alignment through Seed nodes and Alignment Graph methods.

*NetworkBLAST* [49] searches highly connected node groups corresponding to groups of proteins with the same function. NetworkBLAST builds an alignment of the analyzed networks, which is searched for conserved protein complexes. NetworkBLAST assesses the alignment through a suitable Score Function. Each candidate complex is scored by its fit to a protein complex model, which assumes a certain density of interactions within a complex, versus the likelihood that it arises at random.

*NetAligner* [50] presents a strategy to identify evolutionarily conserved interactions, on the basis of consideration that interacting proteins evolve at rates significantly closer than expected by chance. NetAligner computes the alignment through a suitable evolutionary method.

On other hands, GNA algorithms, search for the best mapping that covers all nodes of the input networks by generating a one-to-one node mapping. This strategy considers only the topology of input graphs, leaving out the similarity among small regions. In general, GNAs exploit a two-step schema to build the alignment. At first, the algorithms adopt a cost function, that maximises the node likeness also known as “node preservation”, or the quantity of preserved edges, i.e., “edge preservation”, to estimate the similarity among pair of nodes. Then, they apply an alignment method to find a high-scoring alignment based on the total similarity over the all aligned nodes among all the possible alignments.

For example, *MAGNA* [44] applies a genetic algorithm to build an improved alignment. MAGNA resembles a set of alignments and it chooses the best among them. *MAGNA++* [51] is the MAGNA extension and it ensures to maximize any edge and node conservation measures. MAGNA and MAGNA++ assess the alignment through a suitable genetic algorithm.

*SANA* (Simulated Annealing Network Aligner) [52] uses the Simulated Annealing to build a final alignment, starting with two networks, and an input alignment randomly built or by applying different aligners. It starts to explore a solution space to improve the initial alignment. The solution space consists of alignments neighbors, i.e., alignments that vary only in one or two mappings of individual pairs of aligned nodes concerning the initial alignment. SANA uses neighbours that differ in exactly two mappings to improve the alignment.

*WAVE* [53] uses a seed-and-extend alignment strategy to optimize both node and edge conservation while constructing an alignment. WAVE is used on top of an established node cost function and it leads to a new superior method for global network alignment, favoring conserved edges among nodes with node cost function similar over those with node cost function dissimilarity.

More recently, Malod-Dognin et al. [54] presented UAlign, that associates different alignments built by global network aligner. It aims to overcome the limitation of the global network aligners present in the coverage of built alignments.

There are also different algorithms, such as *GRAAL* [55] and the *GRAAL family* (H-GRAAL [56], MI-GRAAL [57], C-GRAAL [58] and L-GRAAL [59]), that use a special node similarity measure called graphlet degree vectors [60] to build a global alignment. The main characteristic of the graphlet degree vector is the generalization of the node degree, by counting the degree value for all possible connected induced subgraphs up to a certain node number.

*GHOST* [61] uses a novel spectral signature based on the local neighborhood’s topology to measure the topological similarity between subnetworks. The idea behind GHOST consists of building an alignment by combining the novel spectral signature with a seed-and-extend strategy.

*IsoRank* [62] maximizes an alignment quality measure that balances topological and node similarity using a weight factor α. IsoRank assesses the alignment through a suitable Score Function.

*IGLOO* [63] is able to build high functional and topological quality using a seed and extend strategy on the basis of the integration of GNA and LNA strategies.

### 6.3. Pairwise vs. Multiple Network Alignment

The network alignment algorithms can be categorized according to the number of input networks, i.e., pairwise and multiple alignment.

The pairwise network alignment (PNA) takes two networks as input, and it identifies a sub-network with high similarity among the input networks. The multiple network alignment (MNA) builds the alignment among three or more networks and it detects aligned patterns of nodes. PNA and MNA can be classified in a global approach by exploiting a many-to-many node mapping and local approach by exploiting a one-to-one node mapping.

The PNA applies a many-to-many node mapping among the input networks with the goal of finding similar sub-graphs. Otherwise, PNA exploits one-to-one node mapping to search best match by considering the entire input networks. The MNA applies a many-to-many approach to find an alignment as cluster that contains different nodes from one compared network. Otherwise, MNA exploits one-to-one node mapping to build an alignment as a cluster that contain only one node for each compared networ.

Though PNA and MNA have been applied on PPI networks to build the alignment of [2], it has been shown that the alignment constructed with MNA is able to infer more biological knowledge, since its approach is able to detect function information common to different species.

There are different proposed multiple network alignment algorithms in the literature.

*MultiMAGNA++* [64] is a global MNA algorithm based on one-to-one node mapping. MultiMAGNA++ uses a genetic algorithm and introduces a new cost function to derive from the parent nodes a new childhood that allows the construction of subsequent multiple alignments.

*GEDEVO-M* [65] is a global one-to-one MNA algorithm based on an evolutionary algorithm that uses the Graph Edit Distance (GED) as an optimization model for finding the best alignments.

*LocalAli* [66] is a local many-to-many algorithm that uses an evolutionary model to derive an evolutionary tree of networks nodes. LocalAli uses the maximum parsimony evolutionary model to infer the evolutionary tree of networks’ nodes. Then, LocalAli extracts local alignments as conserved modules that have evolved from a single ancestral module.

*IsoRankN* [62] is a global many-to-many algorithm that builds a multiple network alignment by using a spectral partitioning method to find dense and clique clusters.

*SMETANA* [67] is a global many-to-many aligner that computes the node similarities using a probabilistic model and then applies a greedy approach to build a multiple alignment.

*FUSE* [68] is a global one-to-one MNA algorithm that defines node similarities between all pairs of networks by applying a non-negative matrix trifactorization. After that, FUSE applies an approximate maximum weight k-partite matching algorithm to build an alignment between the multiple networks.

*NetCoffee* [69] is a global many-to-many aligner that builds a weighted bipartite graph for every pair of networks and then it applies a simulated annealing approach to construct a multi alignment.

*BEAMS* [70] is a global many-to-many aligner that constructs a graph of node similarities considering protein sequence scores and detects from the graph a set of disjoint cliques that maximizes an alignment quality measure. BEAMS assesses the alignment through a suitable nodes cost function.

Table 4 summarizes the different PPI network alignment algorithms.

## 7. Discussion

The analysis of biological networks, and in particular PPIs and pathways, is an important research topic in modern bioinformatics. Biological networks are gaining more and more attention in the life sciences, and in particular in the omics field.

Several efforts were made by the scientific community to provide NA algorithms for investigating PPIs and pathways. Following analysis of the scientific literature, it arose that there is an imbalance concerning the availability of NA algorithms for PPIs and pathways.

### 7.1. PPI Network Alignment

PPI NA algorithms are very popular, allowing investigation of local and global network characteristics relying on several different methodologies, pursuing the goal to discover conserved interactions across multiple species. This can be performed by comparing conserved substructures across species through network alignment. In fact, the goal of network alignment is to predict the protein functions of an unknown species from known ones. The alignment network algorithms may be grouped into two major categories on the basis of their strategy: Local Network Alignment (LNA), and Global Network Alignment (GNA).

Both LNA and GNA may also be categorized on the basis of the input networks in Pairwise Alignment Algorithms, i.e., algorithms that align two input networks, and Multiple Alignment Algorithms, i.e., algorithms aligning three or more input networks at once.

LNA and GNA are both related to find topological and functional similarities among networks and to transfer biological knowledge from well-studied species to poorly studied ones.

In the recent past many independent GNA and LNA algorithms have been developed that rely on different assumptions and algorithmic solutions. In general, LNA multiple aligners perform better in finding conserved complexes or functional modules than GNA pairwise aligners because they better accept the edge loss in PPI networks, and they even directly or indirectly provide intra-species relationships between proteins.

Moreover, LNA multiple aligners are more suitable for revealing phylogenetic relationships between different organisms. On the other hand, GNA pairwise aligners are suitable for detection of similar topological substructures, as well as functional orthologs.

Many of the available algorithms and tools of PPI NA were extended by using high-performance computing (HPC) paradigms to analyze ever larger networks by exploiting the multi-CPU and multi-cores architectures also available in personal computers. HPC improvements make it possible to analyze huge PPIs in reasonable times, making it possible to provide a solution to previously intractable networks with prohibitive dimensions, e.g., thousands of vertices and hundreds of thousands of edges.

### 7.2. Pathway Network Alignment

Conversely, it is worth noting that all the NA pathway algorithms are suitable to align only metabolic pathways, pointing out a lack of methods that can align signaling and regulatory pathways. The lack of NA pathway algorithms for signaling and regulatory pathways is due to the different meaning of nodes and edges in these two categories of pathways. Indeed, genes, proteins or small molecules in metabolic pathways concern edges because they catalyze reactions, whereas node represent the compound of the catalysis. Differently, nodes in signaling and regulatory pathways represent genes and proteins, and edges represent the interaction among them. The different meaning of biological elements in the three types of pathways make it impossible to use NA algorithm developed to align metabolic pathways for signaling and regulatory pathways, reducing the capability of discovering new pathways or functions. This limitation is not present in PPIs, since all the representation preserves the meaning of nodes and edges. Finally, it is not possible to categorize the available pathway NA algorithm in categories, as is the case for PPI networks.

### 7.3. Pathway and PPIs Database

PPI databases contain information obtained through low-throughput experiments and high-throughput technologies. Pathway databases provide information about the cellular mechanisms’ biological functions through several representation models. This has led to the information heterogeneity between the different databases, making data analysis from multiple databases challenging. To improve the accuracy of network alignment analysis, the researchers need to use both pathway and PPI data. In [45,47], the authors applied the network alignment algorithms to the PPI network of species extracted from the different public databases. The results showed that different alignments were obtained using the networks extracted from the various databases, which is current with the heterogeneity of the information among them. In [71] authors provide a merging signaling pathways method based on protein–protein interaction data. The combined use of pathway and PPI data can help identify similar pathways with the same name or focus on the same biological processes from different data sources in the HPD.

### 7.4. Open Problems in Pathways and PPIs Alignment

It should be noted that the use of graphs has some drawbacks [72]. For example, a problem related to the use of graphs is the lack of supplementary information use about interactions such as spatial and temporal information, kinetics parameters etc. [73].

Another criticism is that PPI and pathway networks are often static, as they do not show the dynamic interaction flow in the cellular over time. Since PPI and pathway networks provide only one viewpoint of the cell’s status, integration with other data types, e.g., temporal and spatial data, is necessary to get a more complete and dynamic picture of cells events.

Furthermore, different models for the PPI networks present some problems limiting the effectiveness of every attempt at comparison due to the missing knowledge of the real interaction map.

Nevertheless, molecular machineries inside cells often involve the interplay of molecules of different types (e.g., genes, proteins and RNA) [74]. Consequently, the use of more complex models such as heterogeneous networks to represent such data has gained the attention of researchers.

PPI and pathway data need to be stored, queried and analyzed. Regarding PPI data storage, the main efforts were devoted to the definition of standards for data exchange, such as HUPO PSI-MI. In addition, each pathway database comes with its own type of data representation formalism. In this heterogeneous landscape, several initiatives were born to try to simplify data exchange, integration and storage, such as BioPAX and SBML. Moreover, both PPI and pathway databases lack a common naming mechanism to uniquely identify interactions. Both databases, discussed above, do not offer refined query mechanisms based on graph manipulation, but they aggregate the only available structured repository for interaction data and permit easy sharing and annotation of such data.

Regarding network alignment algorithms, there are still open problems that have not yet been solved. The first problem is that there is no gold standard ground truth for network alignment across PPI and pathway networks because the nature of biological research is basically a reverse-engineering process due to the unknown mechanisms of evolutionary events. Moreover, there remain inconsistencies and information loss in PPI and pathway data caused by the limitation of existing molecular techniques.

The second issue that needs to be addressed is that there have been more and more large and dense biological networks, and even different types of networks, discovered. This poses problems in terms of execution time and memory consumption. It is obviously a challenge for algorithm design to face them by exploiting HPC, contributing to the resolution of unfeasible problems in a reasonable time without losing accuracy and precision.

## 8. Conclusions

Biological network analysis is a very interesting research field useful for discovering evolutionarily conserved substructures among different species. In fact, different biological data such as pathway and PPI data are recurring to graph formalism by opening new network-based analysis perspectives. In this work, we have tackled the problem of representation and storage, encoding standard and NA algorithms for pathway and PPI networks.

Regarding the representation, directed and undirected graphs are suitable to represent PPI, but they are ineffective to represent complex pathways features such as gene activation/inhibition represented as multiple edges, or reversible catalysis represented through directed and undirected edges. To overcome these limitations, pathways are mainly represented as mixed graphs or hypergraphs. Thus hyper- and mixed graphs allow us to represent complex features of pathways, but on the other hand, many algorithms developed for graphs cannot be directly applied to hypergraphs and mixed graphs. Furthermore, several databases are available for both PPI and pathways. Researchers need to use multiple databases to answer their biological questions; thus, the availability of many databases could contribute to simplify their work. Regardless, the lack of a gold standard for interchange among the available databases, makes the integration, annotation and exchanging process challenging even for bioinformatics. To overcome the lack of standards, many initiatives are emerging with the goal of providing a common standard to harmonize heterogeneity in biological databases. BioPAX is trying to impose itself as a gold standard for integration, exchange, visualization and analysis of biological pathway data. BioPAX supports data exchange, reducing the complexity of interchange between data formats by providing an accepted standard format for pathway data. On the other hand, PPI lacks a standard to uniquely identify interactions.

The network analysis is entangled in precision medicine. Cancer Systems Biology is an example of network analysis playing an essential role in precision medicine. Cancer System Biology is the application of system biology approaches to cancer research. This means studying how intracellular networks of normal cells become cancer cells. The study of these data can determine effective predictive models that can assist the production of new therapies and drugs. For example, systems models can incorporate patient-specific datasets with drug response profiles to define effective personalized treatment options. Cancer systems biology approaches have been applied explicitly to several types of cancer. In addition, whole-genome expression data were integrated with patient outcomes to classify patients’ therapy responses according to gene expression patterns. This allows the identification of specific expression patterns and pathways associated with particular mutations in the tumor. In addition, the altered patterns or drug responses in the cancer cell were also identified. Finally, combining genomic data with mathematical modeling is a step in the right direction toward precision medicine. This approach can potentially improve personalized treatment in the future by understanding the biological functions, and can help study diseases and engineering drugs to fight cancer and other diseases.

Finally, in the literature, many NA algorithms used to analyze pathways and PPI networks are available, even if there is an imbalance in the availability of NA algorithms for PPIs and pathways. This is related to large implementation of PPI NA algorithms for different species with the aim of discovering conserved interactions across multiple species. Instead, NA pathway algorithms enable to compare only metabolic pathways. NA presents a lot of open problems worth further investigation, especially concerning pathway alignment. Although pathways are represented through networks, the development of pathway analysis methodologies is slower compared to PPI, perhaps due to their biological complexity. The development of NA algorithms able to align signaling and regulatory pathway can help researchers to shed light on the pathways involved in disease and detecting which components of the pathway exhibit incorrect behavior. This may lead to more personalized strategies for diagnosing, treating and preventing disease. In addition, the adoption of a unique data format can promote the integration and sharing of pathway data coming from several databases. Moreover, it can support the annotation and integration of pathways with PPI data, since it is becoming increasingly evident that the connection between pathways and diseases is fashioned at multiple interconnected levels.

## Figures and Tables

**Table 1:** The table summarizes the main characteristics of the listed databases. The column #Pat refers to the total number of memorized pathways for each database; PathType provides infromation about the handled pathway type; #P−G provides information about the total number of covered proteins/genes for each database; #Int is the total number of interactions available in each database; organism provides information on the pathway origin organism; Cur specifies how pathway data are handled in each databases. In the table, SMT is the contraction of Signaling, Metabolic and Transduction pathways. MO indicates Multi-Organisms. H is short for Human. LC and EC are short for Literature and Electronic Curation.

Pathway DB	#Pat	PathType	#P−G	#Int	Organism	Cur
Biocarta	254	SMT	1396	×	H,M	LC
BioCyc	20,005	MT	14,544	×	MO	LC
INOH	9606	SMT	22,799	5625	MO	LC
KEGG	551	M	19,618	3568	H	LC
NetPath	36	S	5337	7214	H	LC
PathwayCommons	5772	SMT	18,490	2,424,055	H	EC
Reactome	2553	SMT	31,506	16,017	MO	LC
SMPDB	908	MT	1576	×	H	LC
WikiPathways	3053	SMT	7858	×	MO	LC

**Table 2:** The table summarizes the main characteristics of the listed databases. In the table, the column #Proteinst refers to the total number of memorized proteins for each database; the column #Intereraciton refers to the total number of memorized protein interactions for each database; the column Organism is related to the organisms on which the ppi are inferred; the column Exper Det. records whether the interactions have been experimentally determined.

PPI Networks DB	#Proteinst	#Intereraciton	Organism	Exper. Det.
DIP	28,850	81,923	SC, DM, EC, CE, HS, HP, MM, RN, BT, AT	Yes
BioGRID	2,467,140	1,740,000	SC, DM, EC, CE, HS, HP, MM, RN, BT, AT, SC, SARS-CoV-2, XL	Yes
MINT	27,069	132,249	HS, SC, MM, RN, HP, DM, CE	Yes
IntAct	118,759	1,184,144	HS, SC, MM, RN, EC, DM, CE, HP, SP, BS, SARS-CoV-2	Yes
I2D	687,072	1,279,157	SC, CE, DM, RN, MM, HS	No
STRING	67,592,464	20,052,394,041	HS, MM, AT, SC, EC, CE, RN, DM, BS, PAO1	No

**Table 3:** Pathway alignment algorithms.

Algorithm	one2Many	Many2Many	Method
MP-Align	*√*	×	Maximum weighted bipartite matching algorithm, largest conserved
			sub-hypergraph
PathAligner	*√*	×	Hierarchical alignment
SubMAP	*√*	×	Maximum weight independent set and eigenvalue problem
MetaPathwayHunter	*√*	×	Subtree homeomorphism and weighted assignments in bipartite graphs
MetaPAT	*√*	×	Subgraph homeomorphism
MetNetALigner	*√*	×	Dynamic programming
CAMPways	*√*	×	Constrained alignment

**Table 4:** PPI Network alignment algorithms.

Algorithm	GNA or LNA	PNA or MNA	One-to-One or Many-to-Many	Method
GLAlign	LNA	PNA	Many-to-many	Seed node and alignment graph
NetworkBLAST	LNA	PNA	Many-to-many	Score function
NetAligner	LNA	PNA	Many-to-many	Evolutionary method
AlignNemo	LNA	PNA	Many-to-many	Score function
AlignMCL	LNA	PNA	Many-to-many	Seed node and alignment graph
H-GRAAL	GNA	PNA	One-to-one	Graphlet
MI-GRAAL	GNA	PNA	One-to-one	Graphlet
C-GRAAL	GNA	PNA	One-to-one	Graphlet
L-GRAAL	GNA	PNA	One-to-one	Graphlet
IsoRank	GNA	PNA	One-to-one	Node similarity
GHOST	GNA	PNA	One-to-one	Spectral signature
WAVE	GNA	PNA	One-to-one	Seed and extend strategy
MAGNA	GNA	PNA	One-to-one	Genetic algorithm
MAGNA++	GNA	PNA	One-to-one	Genetic algorithm
SANA	GNA	PNA	One-to-one	Simulated annealing
IGLOO	GNA	PNA	One-to-one	Seed and extend strategy
MultiMAGNA++	GNA	MNA	One-to-one	Genetic algorithm
GEDEVO-M	GNA	MNA	One-to-one	Evolutionary algorithm
IsoRankN	GNA	MNA	Many-to-many	Node similarity
SMETANA	GNA	MNA	Many-to-many	Greedy approach
LocalAli	LNA	MNA	Many-to-many	Maximum parsimony evolutionary model
FUSE	GNA	MNA	One-to-one	Non-negative matrix trifactorization
NetCoffee	GNA	MNA	Many-to-many	Simulated annealing
BEAMS	GNA	MNA	Many-to-many	Node cost function

## Abbreviations

The following abbreviations are used in this manuscript:

PPI	Protein-protein interaction.
NA	Network alignment.
HTO	High-throughput omics.
GWAS	Genome-wide association studies.
NGS	Next-generation sequencing.
Y2H	Yeast two-hybrid.
MS	Mass Spectrometry.
EC number	Enzyme commission.
BioPAX	Biological pathway exchange.
PSI-MI	Proteomics standards initiative—molecular interaction.
INOH	Integrating network objects with hierarchies.
KEGG	Kyoto encyclopedia of genes and genomes.
SMPDB	Small molecule pathway database.
DIP	Database of interacting proteins.
BioGRID	Biological general repository for interaction datasets.
MINT	Molecular interaction database.
I2D	Interologous interaction database.
STRING	Search tool for the retrieval of interacting genes/proteins database.
AT	Arabidopsis thaliana.
BT	Bos taurus.
BS	Bacillus subtilis.
CE	Caenorhabditis elegans.
CS	Chlorocebus sabaeus.
CG	Cricetulus griseus.
DR	Danio rerio.
DM	Drosophila melanogaster.
EC	Escherichia coli.
GG	Gallus gallus.
HS	Homo sapiens.
HP	Helicobacter pylori.
MM	Mus musculus.
RN	Rattus norvegicus.
SC	Saccharomyces cerevisiae.
SP	Schizosaccharomyces pombe.
